# Carbon sequestration potential and physicochemical properties differ between wildfire charcoals and slow-pyrolysis biochars

**DOI:** 10.1038/s41598-017-10455-2

**Published:** 2017-09-11

**Authors:** Cristina Santín, Stefan H. Doerr, Agustin Merino, Thomas D. Bucheli, Rob Bryant, Philippa Ascough, Xiaodong Gao, Caroline A. Masiello

**Affiliations:** 1 Department of Geography, College of Science, Swansea University, Singleton Park, Swansea, SA2 8PP UK; 2 Department of Biosciences, College of Science, Swansea University, Singleton Park, Swansea, SA2 8PP UK; 3 Department of Soil Science and Agricultural Chemistry, University of Santiago de Compostela, 27002 Lugo, Spain; 4 Agroscope, Environmental Analytics, Reckenholzstrasse 191, 8046 Zürich, Switzerland; 5 NERC-RCF, Scottish Universities Environmental Research Centre, Rankine Avenue, Scottish Enterprise Technology Park, East Kilbride, G75 0QF Scotland, UK; 6 Department of Earth, Environment, and Planetary Sciences, Rice University, 6100 Main St MS 126, Houston, Texas 77005 USA

## Abstract

Pyrogenic carbon (PyC), produced naturally (wildfire charcoal) and anthropogenically (biochar), is extensively studied due to its importance in several disciplines, including global climate dynamics, agronomy and paleosciences. Charcoal and biochar are commonly used as analogues for each other to infer respective carbon sequestration potentials, production conditions, and environmental roles and fates. The direct comparability of corresponding natural and anthropogenic PyC, however, has never been tested. Here we compared key physicochemical properties (elemental composition, δ^13^C and PAHs signatures, chemical recalcitrance, density and porosity) and carbon sequestration potentials of PyC materials formed from two identical feedstocks (pine forest floor and wood) under wildfire charring- and slow-pyrolysis conditions. Wildfire charcoals were formed under higher maximum temperatures and oxygen availabilities, but much shorter heating durations than slow-pyrolysis biochars, resulting in differing physicochemical properties. These differences are particularly relevant regarding their respective roles as carbon sinks, as even the wildfire charcoals formed at the highest temperatures had lower carbon sequestration potentials than most slow-pyrolysis biochars. Our results challenge the common notion that natural charcoal and biochar are well suited as proxies for each other, and suggest that biochar’s environmental residence time may be underestimated when based on natural charcoal as a proxy, and vice versa.

## Introduction

Pyrogenic carbon (PyC) is produced both naturally (e.g. charcoal formed during vegetation fires) and anthropogenically (e.g. kiln charcoal and biochar) and comprises a whole range of pyrogenic organic materials from partially charred biomass to soot^1^. Globally vegetation fires burn 300–460 Mha (~4% of the vegetated land surface) every year^2, 3^ and produce 116–385 Tg PyC yr^−1^, which is proposed as a quantitatively significant global carbon (C) sink^1^. Biochar (PyC produced by controlled pyrolysis) is acknowledged as a valuable soil amendment and a C sequestration tool^4^. It has been estimated that global sustainable biochar production could offset up to 12% of current anthropogenic CO_2_ emissions^5^.

PyC materials are therefore the focus of numerous studies which evaluate their potential as long-term atmospheric C sinks and roles in global warming^1, 4, 6–10^. In addition, many other studies address their roles in soil functioning and in other ecosystem properties and services, or use them as paleoenvironmental indicators^11–15^. Biochar is a relatively recent, but already very extensively researched field^16^; however, its use is still not very widespread, partially due to uncertainties regarding its longer-term fate and impacts^17^. The term biochar was coined in the early 2000s^16^ and the monitoring of biochar in the environment, therefore, spans a few years at most, limiting our understanding of its long-term fate and behaviour. To overcome this limitation, charcoal produced during wildfires and in kilns has been widely used as an analogue to give insights into the long-term role of biochar in C sequestration, and also into ecosystem functions such as nutrient cycling, pollutant removal or primary productivity^18–22^. Indeed, the identification of thousands of years-old PyC in Terra pretta soils as the key factor leading to their sustainable use and long-term fertility was the starting point for biochar research^23^.

Conversely, in order to reconstruct the characteristics of past and recent wildfires, properties of wildfire charcoal found in soils and sediments are frequently compared to those of laboratory-generated PyC^24–27^, even when the experimental conditions are more similar to those used for biochar production than to real wildfires^28^. In addition, residence time and C sequestration potential of wildfire PyC in models are often derived from biochar and/or laboratory PyC analysis^1, 4, 10^ also assuming interchangeable properties between these different types of PyC materials.

The C sequestration potential of PyC materials, both natural charcoal and biochar, is mostly estimated using indexes based on different measurements of chemical recalcitrance^4, 29, 30^. Chemical recalcitrance has been directly linked to PyC resistance to degradation^29–32^ and it is thought to be the main control on PyC longevity in mineral soils^33^. PyC chemical recalcitrance is determined by the characteristics of its unburnt precursors (i.e. feedstock), and by the formation conditions under which it is produced^4, 34^. The most frequently used parameter to describe PyC formation conditions is maximum temperature, commonly referred to as ‘Highest Treatment Temperature’ (HTT). Recently, charring intensity (CI) has been suggested as a more appropriate descriptor, as this parameter integrates both heating temperature and duration^35^.

Formation conditions of PyC materials produced naturally and anthropogenically differ. On the one hand, biochar production by slow pyrolysis (which is the most widespread biochar production technology^36^) is carried out under well-controlled stable conditions, in the absence of oxygen, with heating durations of hours and HTTs from 300–700 °C^37^. On the other hand, PyC formation conditions during wildfires (or any type of vegetation fire) are much more heterogeneous, with some degree of atmospheric oxygen availability, and shorter exposure to generally more variable, but typically higher HTTs^26, 29, 38^. In addition, PyC formation via pyrolysis is only one part of the whole biomass burning process, which includes also flaming (combustion of the gaseous vapours) and glowing (oxidation of the solid fuel)^39^. These differences in PyC formation conditions raise critical questions regarding the comparability of biochars and wildfire charcoals. These questions include how exactly production conditions (e.g. HTTs and CIs) differ between charcoals and biochars and to what degree these differences affect their properties and their C sequestration potentials. This study aims to answer these questions by, for the first time, directly comparing wildfire charcoals and slow-pyrolysis biochars produced from the same feedstocks under typical and closely monitored temperature/duration conditions. Slow-pyrolysis biochar has been chosen here instead of biochar produced by other methods (e.g. fast pyrolysis, gasification, hydrothermal carbonization) because it is the focus of most biochar research to date, including field applications^36^.

We first sampled two common wildfire fuels: a forest floor layer (FF, dominated by conifer needles) and dead down wood (DW). We then conducted an experimental crown forest fire, representative of a typical boreal wildfire, and monitored formation conditions (HTTs and CIs values) for charcoal derived from these two fuels (FF and DW). Then, biochar was produced by slow pyrolysis using these two wildfire fuels as feedstock. Lastly, to allow assessment of their comparability, the physicochemical properties of these natural (wildfire charcoals) and anthropogenic (biochar) PyC materials were characterized by elemental, stable C isotope ratios (δ^13^C) and polycyclic aromatic hydrocarbon (PAH) analysis, thermogravimetry-differential scanning calorimetry, solid-state^13^C nuclear magnetic resonance spectroscopy, hydropyrolysis and pycnometry, and their C sequestration potentials determined^4, 29, 30^.

## Results

### PyC production conditions during wildfire vs. slow pyrolysis

During the wildfire, the maximum temperatures (HTTs) recorded at the specific charcoal production sampling locations were highly variable, ranging from 550 °C up to 950 °C (Table 1). This range overlaps with, and extends well beyond, the typical HTTs feedstocks are exposed to in biochar production (300–700 °C^40^) and the range used in this study (350–650 °C; Table 1).

**Table 1:** Chemical and physical parameters analyzed for the forest floor (FF) and dead wood (DW) unburnt feedstocks, wildfire charcoals and slow-pyrolysis biochars.

	HTT (°C)	CI (10^6^ °C s)	Mass loss (%)	C (%)	N (%)	H (%)	O (%)	*δ* ^13^C (‰)	Q3 (%)	R_50_	Degree of aromaticity (%)	SPAC (%)	Skeletal density (g cm^−3^)	Envelope density (g cm^−3^)	Porosity (%)
FF feedstock	n.a.	n.a.	n.a.	39.3	0.87	4.9	38.5	−28.1	8	0.41	8	1.2	n.d.	n.d.	n.d.
FF charcoal #2	550	0.2	78	45.2	1.21	3.5	28.0	−28.9	16	0.46	40	13.5	1.6	n.d.	n.d.
FF charcoal #13	683	0.2	78	53.2	1.29	2.9	27.1	−29.6	11	0.49	59	33.9	1.6	n.d.	n.d.
FF charcoal #20	950	0.1	78	58.2	1.15	3.3	30.6	−28.8	17	0.50	71	40.2	1.5	n.d.	n.d.
FF biochar #1	350	6.6	38	48.8	1.05	3.4	21.4	−29.4	11	0.49	55	23	1.5	0.39	71.8
FF biochar #2	500	11.3	54	57.1	1.12	2.4	14.9	−29.2	16	0.52	89	55.5	1.7	0.43	71.2
FF biochar #3	650	15.9	60	55.7	0.62	1.1	6.4	−29.3	33	0.56	79	94.1	2.0	0.43	77.5
DW feedstock	n.a.	n.a.	n.a.	46.1	0.07	5.4	47.7	−27.5	12	0.40	15	0.1	n.d.	n.d.	n.d.
DW charcoal	796	0.1	84	73.3	0.23	3.0	20.2	−27.9	22	0.55	74	38.1	1.5	0.27	81.2
DW biochar #1	350	6.8	61	68.9	0.16	4.0	24.1	−28.4	13	0.52	71	31.2	1.3	0.29	78.0
DW biochar #2	500	11.6	72	79.3	0.17	2.4	12.4	−28.7	31	0.56	88	69.5	1.6	0.27	83.1
DW biochar #3	650	16.2	75	84.7	0.14	1.7	6.2	−28.7	51	0.60	82	92	2.1	0.28	86.6

^#^Sample number; n.a: not applicable; n.d: not determined.

C sequestration potential according to Harvey *et al*.^30^: Class A: R_50_ < 0.50; Class B: 0.50 ≤ R_50_ < 0.70; Class C: R_50_ > 0.70.

We also calculated, for both wildfire charcoal and slow-pyrolysis biochar, the charring intensity index (CI; Table 1). Pyle *et al*.^35^ suggested using this instead of HTT for a more accurate characterization of pyrolysis conditions, as CI takes into account not only temperature but also heating duration. Despite the higher HTTs recorded for wildfire charcoals, their CI values were over an magnitude lower than those recorded for biochar. Wildfire charcoal CIs ranged from 0.1 to 0.2 × 10^6^ °C s, whereas CIs for biochars spanned from 6.6 to 16.2 × 10^6^ °C s (Table 1). Our biochar CIs are higher than those reported by Pyle *et al*.^35^ (1.8–10.2 × 10^6^ °C s) due to slower heating and cooling rates in the present study. However, even the lowest CIs in Pyle *et al*.^35^ are also one order of magnitude higher than those obtained here for wildfire charcoal (Table 1).

**Figure 1:**
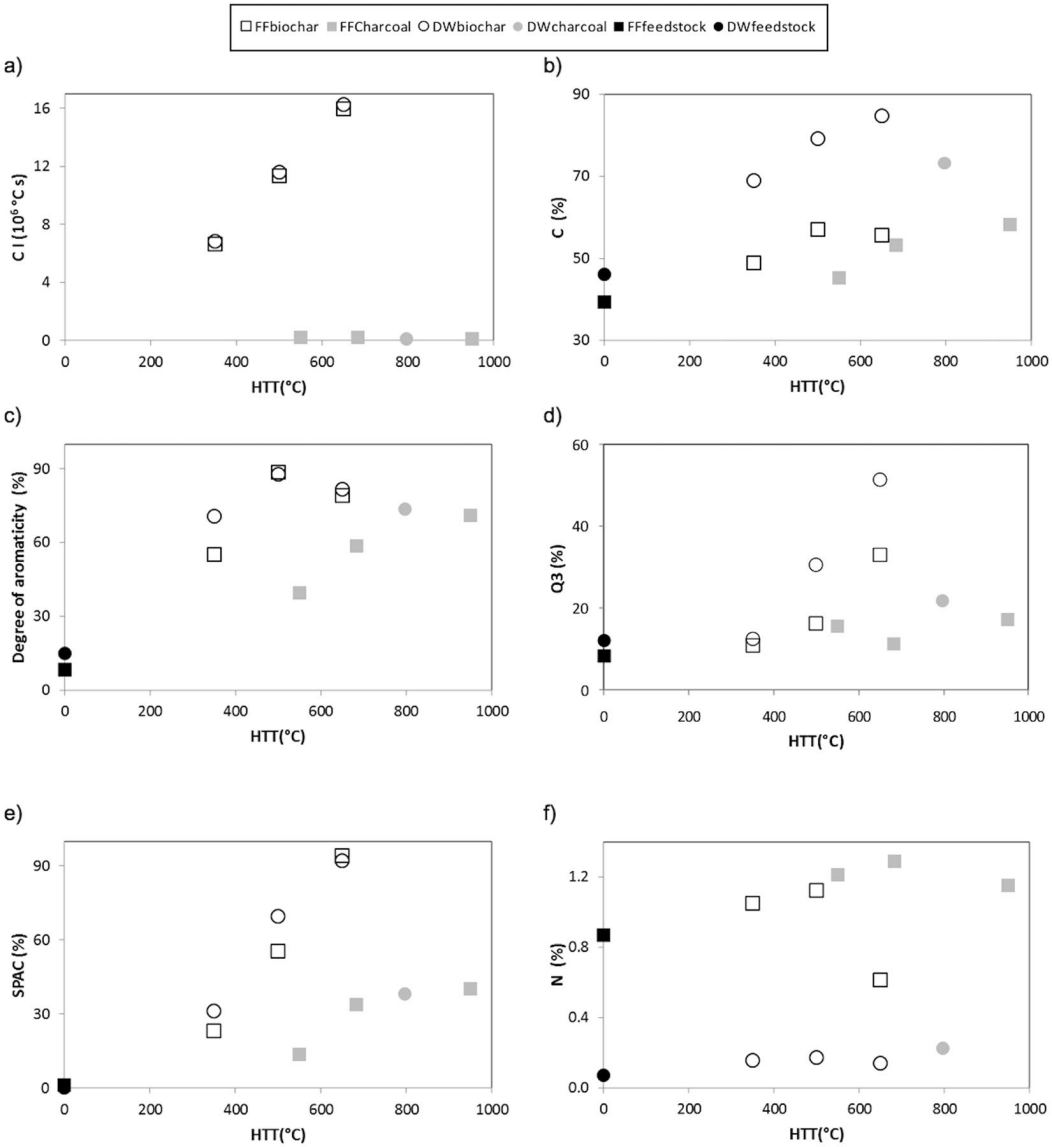
(**a**) Relationship between monitored wildfire and slow-pyrolysis maximum temperature (HTT) and charring intensity (CI) for the studied PyC samples; Fig. 1b–e Relationships between HTT and selected chemical properties of the studied PyC samples and their feedstocks. FF: forest floor; DW: dead wood. For other parameters and specific values see Table 1.

For biochar samples, higher CIs were associated with higher HTTs, but this relationship does not hold for the wildfire charcoal samples (Fig. 1a). All wildfire charcoal samples display very similar CIs despite the broad range of HTTs to which they were subjected (Fig. 1a). This suggests that CI may be less suitable than HTT as a descriptor of the variation of wildfire charcoal’s chemical and physical properties with formation conditions. For our combined wildfire charcoal-biochar dataset only HTT is, therefore, used below to examine trends.

### Chemical transformations of PyC materials during wildfire vs. slow pyrolysis

Wildfire charring and slow pyrolysis led to similar chemical transformations of the two feedstocks analysed (forest floor, FF; and dead wood, DW), but to varying magnitudes. For the two feedstocks, both wildfire charring and slow pyrolysis led to notable mass loss, C enrichments, O and H losses, slight decrease of the δ^13^ C signature and substantial increases in chemical recalcitrance, as indicated by raised thermal recalcitrance indexes (Q3%, R_50_) degree of aromaticity, and stable polycyclic aromatic carbon (SPAC) content (Table 1). The N content was enriched in all of the PyC materials, except for the FF biochar formed at 650 °C (Table 1).

Increasing HTTs resulted, both for wildfire and slow pyrolysis, in enhanced chemical recalcitrance, as indicated by increasing C%, Q3%, R_50_, degrees of aromaticity, and SPAC contents (Fig. 1b–e and Table 1). However, the chemical recalcitrance of wildfire charcoal subjected to high HTTs was more similar to biochar formed under low HTTs rather than to high HTT biochar (Fig. 1b–e and Table 1). For a given HTT, the recalcitrance indicators were mostly more similar between biochar samples obtained from DW and FF than between biochar and wildfire charcoal derived from the same feedstock (see HTTs 500–550 and 650–700 °C in Fig. 1c–e). For biochar, the differences in recalcitrance, measured by SPAC% and degree of aromaticity, between the two feedstocks diminished with increasing HTTs, in line with previous observations^41, 42^ (Fig. 1c and e).

The N% showed a decrease at the highest HTTs for both formation processes (Fig. 1f). Wildfire charring led to higher N% than slow pyrolysis (Fig. 1f), which could be partially due to a substantial inorganic N fraction in the natural PyC materials^43^. For the δ^13^C signature, no trends were observed with increasing HTT, as the values were very similar along the range of wildfire charring/pyrolysis conditions studied, all slightly more negative than the unburnt feedstocks (Table 1), which can be explained by the loss of isotopically heavy cellulose^44^.

**Figure 2:**
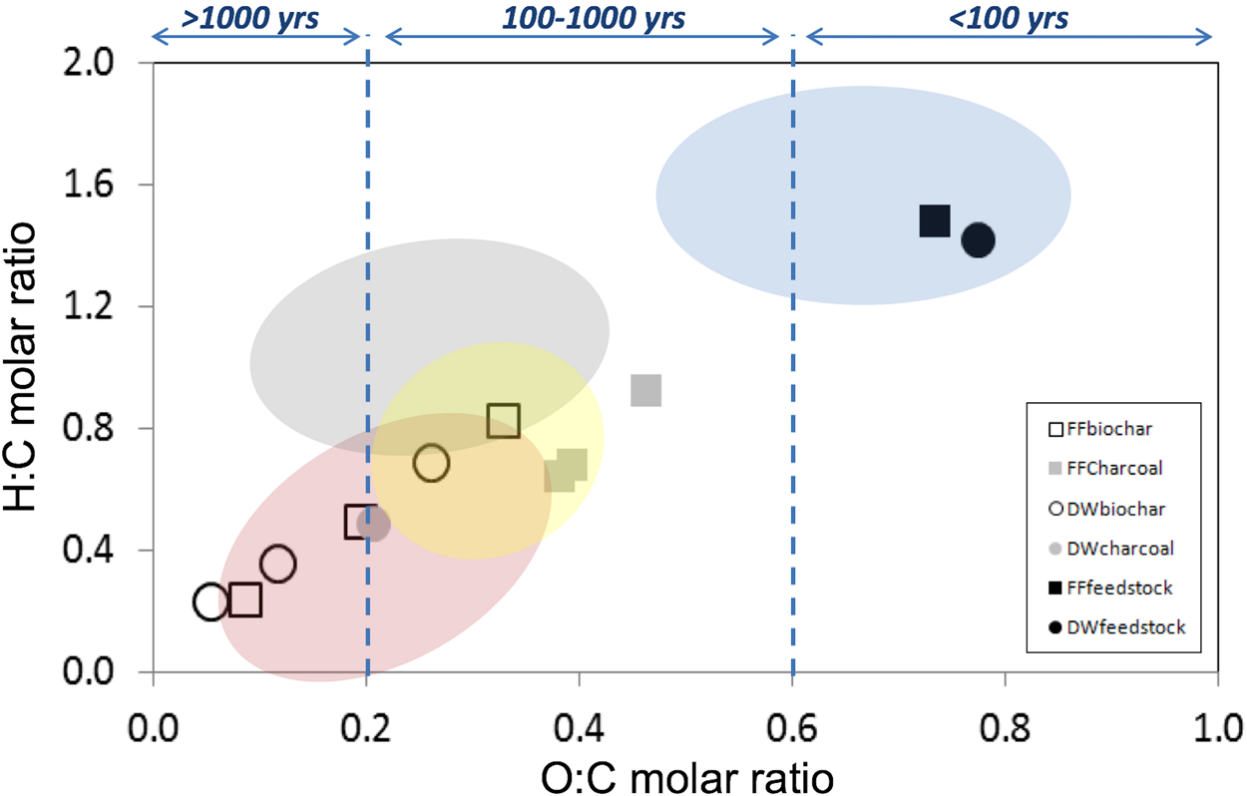
Van-krevelen diagram with H:C and O:C molar ratios for dead wood (DW) and forest floor (FF) feedstock, biochars and charcoals. The areas in blue, grey, pink and yellow indicate typical values for feedstocks, hydrochars, biochars and wildfire charcoals, respectively^25, 37, 47^. The dotted lines in the X axis mark the half-lives ranges (>1000 yrs; 100–1000 yrs; and <100 yrs) according to Spokas^29^.

The H:C and O:C molar ratios serve as indicators of the degree of carbonization, reflecting condensation and oxidation, respectively^45^. H:C and O:C molar ratios for our biochars and charcoals (Fig. 2) are in line with previously reported values^25, 29, 37, 45–47^. The negative linear relationship found by Spokas^29^ between O:C molar ratios and HTTs for biochars also holds for our biochar samples (Fig. 2). However, for wildfire charcoal, comparable HTTs resulted in higher O:C and H:C ratios, which indicates less condensation and higher oxidation (Fig. 2, Table 1).

### Carbon sequestration potential of PyC materials formed during wildfire vs. slow pyrolysis

According to the classification of Spokas^29^, all our wildfire charcoals, irrespective of their HTTs and feedstocks, have expected half-lives of 100–1000 yrs, whereas only the biochars formed at 350 °C belong to that class, and all the biochars formed at 500 and 650 °C have half-lives of >1000 yrs (Fig. 2).

According to the classification of Harvey *et al*.^30^, the FF charcoals formed at the lowest HTTs (<700 °C) have low sequestration potentials, comparable to uncharred biomass (Class A, R_50_ <0.50^30^) (Table 1). Also the FF biochar formed at the lowest HTT belongs to this Class A (350 °C; Table 1). All the other biochars analysed fit into Class B (0.50 ≤ R_50_ < 0.70; Table 1), and, therefore, have ‘intermediate’ C sequestration potentials. Only the charcoals formed at the highest HTTs belong to this Class B (950 °C for FF and ~800 °C for DW, Table 1). None of the PyC materials studied here are within Class C (R_50_ > 0.70), which has the highest C sequestration potential, comparable to graphite and soot^30^.

Bird *et al*.^4^ suggest a 3 pool model for the PyC degradation continuum: a labile (half-life of weeks to months), an intermediate semi-labile pool (half-life of years to centuries) and a stable pool or SPAC (half-life of centuries to millennia). In their model, the proportions of these pools are determined by the HTTs, with PyC formed <400 °C dominated by the intermediate semi-labile pool and PyC formed >600 °C mostly consisting of SPAC. Our biochar data agree with this model, and therefore, most of the biochar formed at high HTT is expected to have half-lives of centuries to millennia (Table 1); however, this is not the case for our wildfire charcoal where the SPAC content did increase at higher HTTs, but was always ≤40% (Table 1). Hence, only a small proportion (<40%) of the wildfire charcoal will have a half-life of centuries to millennia.

Therefore, even if the original material (feedstock) does affect the C sequestration potential of the studied samples, with FF PyC materials being less resistant to degradation than comparable DW PyC, the main driver seems to be the formation process, with higher C sequestration potentials estimated for slow pyrolysis biochars than for wildfire charcoals.

### Physical transformations of PyC materials during wildfire vs. slow pyrolysis

Skeletal density reflects the density of a material without considering internal pores, and presents an upper limit for the environmental density of the material. All wildfire charcoal samples had skeletal densities ≤1.6 g cm^−3^. The biochars formed at the highest HTTs, in contrast, showed skeletal densities values up to 2.1 g cm^−3^ (Table 1). The difference between biochar and wildfire charcoal skeletal densities at similar HTTs (Table 1) likely occurs because the biochars’ exposure to sustained, stable, high temperatures leads to more homogeneous contraction of cell wall structures and more thorough structural condensation at the nanometer scale^48^.

Our biochars followed the previously reported trend of increasing skeletal density with increasing HTTs^46, 49^, although this trend may be a result of the conflation of HTT and CI. For our samples, biochar skeletal density increased to>1.6 g cm^−3^ for HTTs ≥500 °C and CI >7 × 10^6^ °C s. The fact that the FF wildfire charcoals did not show the trend of increasing skeletal density with increasing HTTs suggests that shifts in skeletal density indeed require sustained exposure to high temperatures.

Regarding the envelope density, as with previous studies^49^, there was little effect of HTT. Envelope density was always <1 g cm^−3^, reflecting a porosity ranging from 71–87% (Table 1). These porosities are high enough, and envelope densities low enough, that without additional ballast these materials should be highly landscape-mobile. Our results are consistent with previous reports suggesting both biochar and natural charcoals will initially float, and then, when water-logged, will sink^50^.

### PAHs concentration and distribution in PyC materials formed during wildfire vs. slow pyrolysis

**Figure 3:**
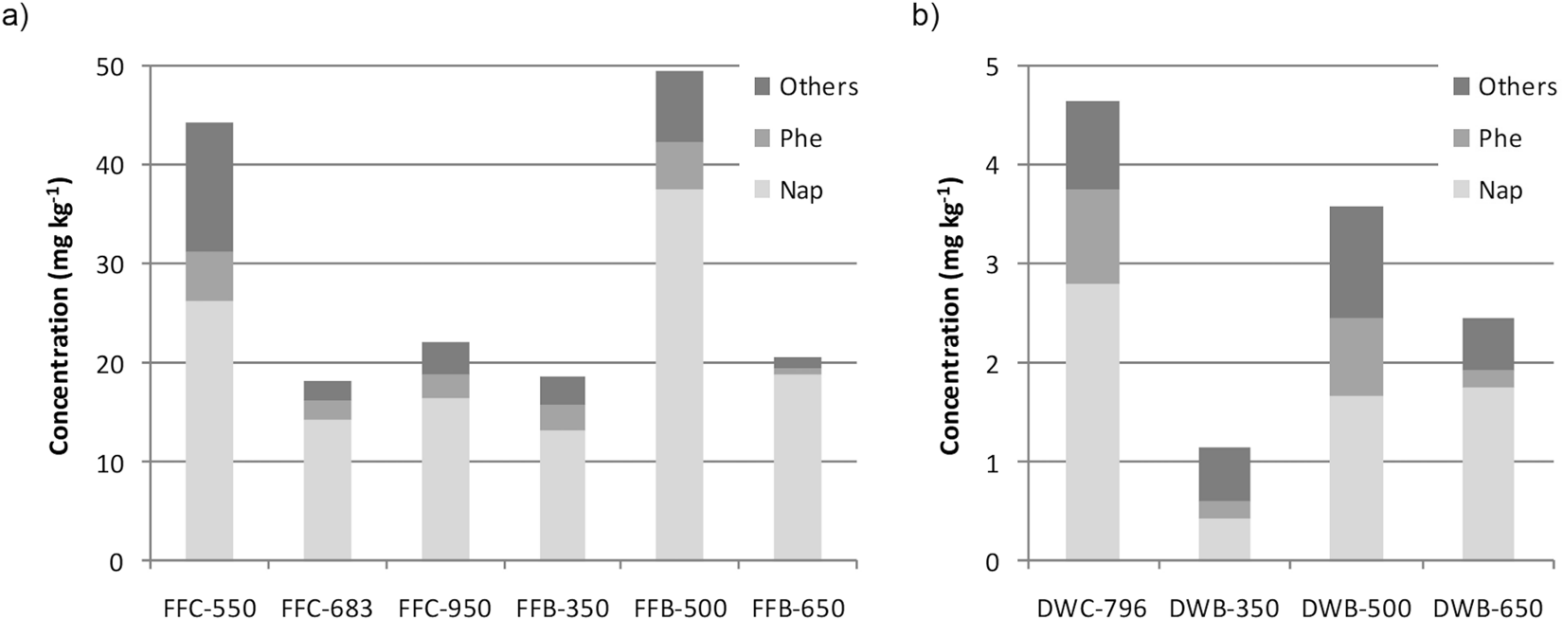
PAHs concentrations (mg kg^−1^ dry sample) divided in naphthalene (Nap), phenanthrene (Phe), and the sum of the other 14 US EPA PAHs (Others) for (**a**) forest floor charcoal (FF-C) and biochar (FF-C); (**b**) dead wood charcoal (DW-C) and biochar (DW-B). The numbers in the X axis indicate the HTT of each sample. Note the different scales for the verticle axis between the two graphs.

Σ16 PAHs concentration was the only parameter analysed where feedstock type, and not formation process, was clearly the dominant driver. FF PyC materials had concentrations of Σ16 PAHs 10 times higher (18–50 mg kg^-1^) than the DW PyC materials (1–5 mg kg^−1^), irrespective of whether they were formed under wildfire or slow pyrolysis conditions (Fig. 3). DW PyC materials did not have Σ16 PAHs concentrations above the threshold set for basic grade biochars according to the European Biochar Certificate (12 mg kg^−1^)^40^, but all FF PyC materials were well above this (Fig. 3). For the FF feedstock, HTTs ~500 °C yielded the highest Σ16 PAHs concentrations, both for biochar and charcoal (Fig. 3a). This is in agreement with previous observations for biochar^51, 52^. For the DW feedstock, the sample pyrolysed at 500 °C also exhibited the highest Σ16 PAHs concentration among the biochar samples, but the only wildfire sample analysed (HTT ~800 °C) showed the overall highest Σ16 PAHs concentration (Fig. 3).

Regarding the distribution of PAHs, all samples showed a clear predominance of light PAHs (2 and 3 rings) (Fig. 3), which is common not only for biochar but also for wildfire ash, charred vegetation and burnt soils^53–55^. Naphthalene (2 rings) and phenanthrene (3 rings) dominated in all samples, but there were higher proportions of naphthalene and lower ones of phenanthrene in FF PyC materials (60–91% and 4–15%, respectively) compared with DW PyC materials (38–72% and 6–22%, respectively) (Table S1). High proportions of naphthalene have been also reported for charred bark (83%) and litter (67%) after a wildfire in a Korean pine forest^55^. Only for the biochar samples, a rise in the proportion of naphthalene was observed with increasing HTT for both FF and DW (Fig. 3). This trend for biochar has been previously documented^51^.

## Discussion

This study directly compares, for the first time, the physicochemical properties of wildfire charcoals and slow-pyrolysis biochars produced from identical feedstocks and under known conditions. Our results challenge the common notion that natural charcoal is a suitable proxy for biochar and vice versa^18–22^. This is especially relevant regarding their respective potentials as C sinks, as even the wildfire charcoals formed at the highest temperatures had lower C sequestration potentials than most biochars produced by slow pyrolysis. For wildfire charcoal, Bird *et al*.^4^ suggest around 10% labile C, 40% semi-labile C, and 50% SPAC, based on the assumption that fires achieve average temperatures of ∼500 °C. Our results suggest that even if their assumed HTT is too low to represent wildfire conditions (Table 1), their SPAC content is an overestimation. Even the wildfire charcoal samples formed at the highest HTTs (up to 950 °C) show SPAC values of <40% (Table 1).

Our results agree with previous findings that feedstock properties influence PyC characteristics^34^ but point to formation process (wildfire charring vs. slow pyrolysis) as being even more important (with the exception of PAHs concentration and distribution, that will be discussed below). They also support the concept that PyC materials produced under higher HTTs have associated higher C sequestration potentials and longer half-lives^26, 29, 30, 38, 56, 57^, irrespective of whether they were formed during wildfires or by slow pyrolysis. Nevertheless, the specific values differed greatly between slow-pyrolysis biochars and wildfire charcoals. Consequently, even if HTT is a useful descriptor of PyC formation conditions and C sequestration potential for both pyrolysis and wildfire charring, its specific effects are not comparable for these two processes.

Why do the effects of HTT differ between wildfire charring and slow pyrolysis? One of the main differences between these two PyC formation processes is the heating duration (in the present study represented by CI). CI can be therefore partially responsible for the substantial differences found in wildfire charcoal vs biochar characteristics, with, in principle, lower CIs resulting in lower chemical recalcitrance for wildfire charcoals. Pyle *et al*.^35^ identified CI ~5 as the threshold where the degree of condensation in biochar starts to increase. However, this threshold does not hold for wildfire charcoal, where CI is always <0.3, but even with these low CI values, the degree of condensation (measured here as SPAC%) increases substantially with increasing HTT (Table 1). Further evidence that variability of CI alone does not explain the differences found here between wildfire charcoal and biochar emerges from comparing our wildfire charcoal samples to fast-pyrolysis biochar (with much shorter heating durations than slow pyrolysis, and therefore, more similar CIs to wildfire conditions). Cotrufo *et al*.^58^ found that the SPAC content in fast pyrolysis biochar [<5 min; 400–700 °C] was actually higher (82%) than in slow pyrolysis biochar (63%) (both derived from pine wood). Brewer *et al*.^59^ found higher aromaticity for slow- vs. fast-pyrolysis biochars (94% vs. 81–83%), but similar cluster sizes in both (7–8 rings). These results indicate than slow- and fast-pyrolysis biochars, even with very different CIs, are more similar between themselves and more recalcitrant than the wildfire charcoal samples examined here. Therefore other factors, besides HTT and CI, need to be explored to fully understand the observed differences. We suggest oxygen availability as another major driver of these differences. Recent work has shown that for slow-pyrolysis biochars, even slightly oxidizing conditions (5% O_2_ atmosphere) can speed up the pyrolysis process substantially^60^. This could help explain the PyC condensation reactions during wildfire charring even with very short heating durations (i.e. CI  <<5). Unfortunately, no instruments currently exist that allow quantifying relevant O_2_ availability data during wildfire charcoal production.

As mentioned above, PAHs concentration and distribution are the only PyC characteristics studied here that are more influenced by feedstock type (FF vs. DW) than by the specific PyC formation process (wildfire charring vs. slow pyrolysis). Irrespective of the formation process, the FF PyC materials presented much higher total PAHs concentrations than the DW PyC materials (Fig. 3). This may be partially due to the chemical properties of the different feedstocks. For example, lower PAHs concentrations in wood biochar than in grass and straw biochars have been reported^52, 61^. However, also feedstock physical characteristics, and, specifically, feedstock arrangement, could play a major role. There are two mechanisms for PAHs formation during combustion and pyrolysis: pyrosynthesis (gas phase reactions) and transformation (solid phase reactions)^40^. The PAHs detected by the analytical method applied here are mostly formed via pyrosynthesis, and, therefore, the resulting PAH concentrations largely depends on whether or not gas-phase pyrosynthesized PAHs have the opportunity to recondensate on solid residual PyC^61–63^. We speculate that the small cylindrical pieces of DW would have most of their surface exposed and, therefore, available for releasing PAHs in the gas phase (Fig. S1); however, the fine fuel particles in the FF (mostly needles) would have, in principle, higher individual surface areas but, the tighter arrangement of these particles within the FF layer (Fig. S1) could have promoted PAHs in the gas phase to get entrapped and recondensated within the fuel bed.

## Conclusions

Here we compare, for the first time, PyC materials formed from the same feedstocks under monitored wildfire charring and slow pyrolysis conditions. Our results challenge the notion of the suitability of wildfire charcoal as a proxy for biochar, and vice versa. In addition, we also urge for caution when producing charcoals in the laboratory and using their characteristics for inferring those produced under wildfire conditions (e.g. temperature formation information)^24–27^. The longer heating durations and limited availability of oxygen, typically used in the laboratory, will result in PyC with characteristics unlikely to represent those generated under similar HTTs during wildfires. This also has important implications for paleoenvironmental studies where, for example, fire regimes and severities are commonly inferred by comparing natural charcoal samples with laboratory charcoals^12, 28, 64^. In order to simulate wildfire conditions as realistically as possible, open flaming fires with relatively short duration should be used instead of longer heating times using muffle furnaces and ovens, as the latter more closely resemble slow pyrolysis than realistic wildfire conditions. Moreover, in its current form, the CI index^35^ is not an adequate descriptor for wildfire charring conditions, and, therefore, future research on optimization of this index is recommended.

We have demonstrated that, even when generated from the same feedstock and under similar HTT, the physicochemical properties of charcoal and biochar are very different and, therefore, their fates and C sequestration potentials may also differ substantially. The observed differences could also translate in different respective roles in soil functioning and other ecosystem properties. The only exception were PAHs concentrations and distributions, which were affected more by the type of feedstock rather than by the process of formation *per se*. Our results support the conceptual model for natural PyC (wildfire charcoal) as heat-altered biopolymers dominated by small polyaromatic clusters^26, 65, 66^ instead of being composed of highly condensed aromatic structures, which would be more typical of slow-pyrolysis biochars produced at medium and high HTTs^48^. This has important implications for the potential role of natural PyC as a C sink, as even the wildfire charcoal formed at the highest HTTs will have more limited C sequestration potential compared to most biochar produced by slow pyrolysis. Estimated residence times of natural PyC in current models and budgets are mostly based on data from biochar and/or laboratory charcoals^1, 4, 10^, and therefore, they may not be as realistic as previously assumed.

## Methods

### Materials: PyC and feedstock samples

Wildfire charcoal samples were collected following an experimental high-intensity crowning forest fire in boreal Canada, conducted to replicate a typical boreal wildfire (June, 2012, Fort Providence, Northwest Territories; Fig. S1). The plot burnt was a mature jack pine (*Pinus banksiana*) stand. Before the fire, samples of forest floor (FF, n = 10) and 0.5–2 cm diameter dead down wood pieces of jack pine (DW, n = 8) were collected. FF is defined here as the surface organic soil layer, composed of needles, mosses, lichens, fermented litter and humidified organic material^38^. Thermocouples (n = 27, Lascar, Easylog, USA) were placed at the FF (~1cm depth) to record temperatures during the fire in this organic soil layer^67^. The morning after the fire, at each of the 27 points instrumented with thermocouples, the top charred layer of the FF was sampled (1.3 ± 0.6 cm depth)^38^. For the present study, 3 samples of this charred FF layer (i.e. FF charcoal) spanning a range of maximum temperatures (HTTs) recorded during the fire were chosen: one sample with a HTT of 550 °C, another with a HTT of 683 °C and the last one with an associated HTT of 950 °C (FF samples numbers 2, 13 and 20 respectively in Santín *et al*.^38^). 10 pieces of completely charred DW (0.5–2 cm diameter) were also collected within the experimental plot and, for this study, a single composite sample was generated and used (i.e. DW charcoal). Further details of the study site, experimental design and fire are given in Santín *et al*.^6, 38^.

For biochar production, the feedstock (Fig. S1) consisted of FF and DW samples collected at the same site before the forest fire as explained above. A composite FF feedstock sample combining samples from 10 different sampling points was generated. DW feedstock consisted of 8 pieces of ~1.5–2 cm length (0.5–2 cm diameter) for each pyrolysis treatment. All feedstock material was dried at 110 °C to constant weight and not ground before pyrolysis. FF and DW biochars were produced by slow pyrolysis at three HTTs: 350, 500 and 650 °C. These HTTs cover the typical range used in slow pyrolysis^37^. A Vecstar VTF 7 tube furnace flushed with N_2_ gas, with a heating rate of ~10 °C min^−1^ and a hold time of 2 hours at the specific HTT was used as in Woolf 2011^68^, at Swansea University, UK.

### Monitoring and calculation of PyC production conditions

For the FF charcoal samples, temperature-duration records were obtained from the corresponding thermocouples, which recorded temperatures every second during the experimental wildfire (see Materials). For the composite DW charcoal sample, thermocouples were not set in down wood pieces during the 2012 experimental forest fire. As an approximation, temperature-duration records from a comparable experimental high-intensity crowning fire (June, 2015) in the same study area were used. Fire characteristics were very similar for both fires: during the 2012 fire, the average HTT at the FF surface was 745 °C (range 550–976 °C) and the average heating duration of T > 300 °C was 180 s (range 65–364 s)^38^; during the 2015 fire, the average HTT at the FF surface was 807 °C (range 610–984 °C) and the average heating duration of T > 300 °C was 193 s (range 80–370 s) (Doerr & Santin, unpublished data). Before the 2015 fire, thermocouples were attached to dead down wood pieces of jack pine (length ~5 cm, diameter ~2 cm, n = 12). The median value from these 12 samples is used, as an approximation, for calculating HTT and CI for the one composite DW wildfire charcoal sample analysed here (Table 1).

For FF and DW biochar samples, temperature-duration data were obtained via the thermocouple of the Vecstar VTF 7 tube furnace, which allowed recording of the temperature every 5 seconds during the pyrolysis process, including the heating and cooling phases.

HTT was obtained for all wildfire charcoal and biochar samples directly from their corresponding temperature-duration records. Charring intensity (CI) was calculated also from the temperature-duration records T(t) following Pyle *et al*.^35^ as in Equation 1:

1 $${\rm{CI}}={\int }_{{\rm{t0}}}^{{\rm{tt}}}{\rm{T}}\,({\rm{t}}){\rm{dt}}$$

where T(t0) is the time at which the rising T(t) record reached 200 °C and t_t_, is the time when the declining T(t) reached 200 °C using a reference T of 0 °C^35^.

### Elemental and δ^13^C analysis and molar ratios calculation

Total C, nitrogen (N) and hydrogen (H) contents (%) were determined using a LECO elemental analyser, and the oxygen (O) content (%) with a FISONS elemental analyser at the University of Santiago de Compostela (Spain). Stable C isotope ratios (δ^13^C) were determined in duplicate at Swansea University (UK) using an ANCA GSL elemental analyser interfaced with a Sercon 20/20 mass spectrometer^38^.

H:C and O:C molar ratios were calculated for feedstocks and PyC materials and plotted in a Van-krevelen diagram (Fig. 2). From the O:C ratio values, half-life (t_1/2_) ranges were estimated according to the classification of Spokas^16^: O:C < 0.2 = t_1/2_ > 1000 yrs; O:C 0.2–0.6 = 100 yrs < t_1/2 < _1000 yrs; O:C > 0.6 = t_1/2_ < 100 yrs (Fig. 2).

### Thermogravimetry - Differential scanning calorimetry (TG-DSC)

TG-DSC thermographs were obtained using a Mettler Toledo instrument at the University of Santiago de Compostela (Spain). Four mg of sample were placed in aluminium pans under dry air (under O_2_ flux; flow rate, 50 mL ^−1^) at a scanning rate of 10 °C min^−1^. The temperature ranged between 50 and 600 °C. Samples of indium (mp: 156.6 °C) were used to calibrate the calorimeter. Samples were analysed in duplicate. The area under each DSC thermograph was divided into three temperature regions representing different levels of resistance to thermal oxidation^69^: labile organic matter, mainly comprising carbohydrates, proteins and other labile aliphatic compounds (150 < T_1_ < 375 °C); recalcitrant organic matter, such as lignin or other polyphenols (375 < T_2_ < 475 °C); and highly recalcitrant organic matter, such as polycondensed aromatic forms (475 < T_3_ < 600 °C). The resulting partial heats of combustion representing these three regions were calculated as Q1, Q2 and Q3, respectively. In this study, Q3 (%) is used as an indicator of thermally recalcitrant organic matter which has also proved to be more resistant to microbial degradation^70^. From the TG thermographs, the recalcitrance index R_50_^30^ was calculated as: R_50,x_ = T_50,x_/T_50,graphite_; where T_50,X_ is the temperature value at which 50% of the total mass of the *x* sample is lost, and T_50,graphite_ the temperature value at which 50% of the total mass of graphite is lost. For the graphite, Alfa Aesar graphite powder, 99.9% purity was used, and the TG-DSC cut-off temperature was 1100 °C. T_50,graphite_ was 823 °C. From the R_50_ values, the studied materials can be classified into the following recalcitrance/C sequestration potential classes according to Harvey *et al*.^30^: Class A: R_50_ < 0.50; Class B: 0.50 ≤ R_50_ < 0.70; Class C: R_50_ > 0.70 (Table 1).

### Solid state^13^C Nuclear Magnetic Resonance spectroscopy

^13^C cross polarization-magic angle spinning (CP-MAS) Nuclear Magnetic Resonance (NMR) spectroscopy was performed using an Agilent Varian VNMRS-500-WB spectrometer at the University of Santiago de Compostela (Spain), following the same operational procedures as in Santin *et al*.^38^. The NMR spectra were processed using the MestreNova software 8.1.0 (Mestrelab Research Inc, USC). For quantification, the spectra were divided into four regions representing different chemical environments of the^13^C nucleus: alkyl C (0–45 ppm), O-alkyl C (45–110 ppm), olefinic and aromatic C (110–160 ppm), and carbonyl C (160–210 ppm). The degree of aromaticity (%) was calculated as: aromatic-C*100/(alkyl C +  + O-alkyl-C + aromatic-C)^71^.

### Hydropyrolysis

The fraction of stable polycyclic aromatic C (SPAC) was determined by hydropyrolysis (HyPy) at the Scottish Universities Environmental Research Centre (UK), according to Ascough *et al*.^72^. Briefly, 100–300 mg of sample was loaded with a dispersed sulphided molybdenum (Mo) catalyst. The catalyst was added at 5% by weight, and then the catalyst-loaded samples were heated and agitated gently to coat the sample with catalyst, before drying under air at 70 °C. For analysis, samples were placed into the reactor of the HyPy rig (Strata Technology Ltd., Nottingham, UK), and pyrolysed at 550 °C under a hydrogen pressure of 150 bar to reductively remove all non-SPAC organic C. This was achieved by using a sweep gas flow of 5 L min^−1^ (ATP) during pyrolysis, which removed non-SPAC pyrolysis products (e.g. polycyclic aromatic hydrocarbons <7 aromatic rings) from the sample. Weight loss during HyPy was recorded. The SPAC content (%) of samples was derived by comparing the organic carbon (OC) content of the catalyst loaded samples with their hypy residues (defined as SPAC/OC%). The OC content of samples before and after HyPy was obtained by elemental analysis on a Costech elemental analyser (EA). The SPAC content of the sample was then calculated via Equation 2 below:

2 $$SPAC\,( \% )=\frac{Residual\,OC\,(mg\,C\,in\,hypy\,residue\,including\,spent\,catalyst)\ast 100}{Initial\,OC\,(mg\,C\,\,n\,sample\,including\,catalyst)}$$

### Pycnometry analysis

Skeletal density, envelope density and porosity were measured in duplicate following Brewer *et al*.^49^ at Rice University (US). Briefly, skeletal density (i.e. the density of the solid frame of the sample, which includes any pores not accessible to helium gas) was measured using an AccuPyc II 1340 gas displacement analyser fitted with a 1 cm^3^ chamber (Micromeritics, Norcross, GA). Envelope density (i.e. sample mass divided by the total sample volume, as if an envelope was placed around each individual particle) was measured using a GeoPyc 1360 envelope density analyser (Micromeritics, Norcross, GA) with 120 μm particles DryFlo as displacement medium. Porosity (%) was then calculated as (1-(envelope density/skeletal density)) * 100. Envelope density measurements have to be performed on unground samples. For FF charcoal, only ground samples were available and, therefore, envelope density and porosity could not be determined.

### Polycyclic Aromatic Hydrocarbons analysis

Concentrations of the 16 PAHs listed as priority pollutants by the US Environmental Protection Agency (EPA)^73^ were determined according to Hilber *et al*.^74^ at Agroscope (Switzerland). Briefly, after addition of deuterated internal analogues of each of the 16 US EPA PAHs, 0.5–1.2 g of sample were extracted for 36 h in Soxhlet with 100% toluene. After addition of a keeper (Isooctane, 1 mL), sample extracts were concentrated to 1 mL with the Syncore Analyst system (Büchi, Flawil, Switzerland). Separation and detection was done by gas chromatography mass spectrometry (Agilent GC 6890N-MS 5973i), and quantification was performed using the internal standard method described in Bucheli *et al*.^75^.

### Data Availability

The datasets generated during and/or analysed during the current study are available from the corresponding author on reasonable request.

## Electronic supplementary material

Supplementary Information
