# Supplementary material for: Carbon sequestration potential and physicochemical properties differ between wildfire charcoals and slow-pyrolysis biochars
Source: Sci Rep. 2017 Sep 11;7:11233. doi: 10.1038/s41598-017-10455-2 (PMC5594023; doi:10.1038/s41598-017-10455-2)
Supplement: Supplementary file 1 — Supplementary Information [file 41598_2017_10455_MOESM1_ESM.pdf]

Carbon sequestration potential and physicochemical properties differ between wildfire charcoals and slow-pyrolysis biochars

Cristina Santín[1*], Stefan H. Doerr[1], Agustin Merino[2], Thomas D. Bucheli[3], Rob Bryant[1], Philippa Ascough[4], Xiaodong Gao[5], Caroline A. Masiello[5]

[1]College of Science, Swansea University, Singleton Park, Swansea SA2 8PP, UK

[2]Department of Soil Science and Agricultural Chemistry, University of Santiago de Compostela, 27002 Lugo, Spain

[3]Agroscope, Environmental Analytics, Reckenholzstrasse 191, 8046 Zürich, Switzerland

[4]NERC-RCF, Scottish Universities Environmental Research Centre, Rankine Avenue, Scottish Enterprise Technology Park, East Kilbride, G75 0QF, Scotland, UK

[5]Department of Earth, Environment, and Planetary Sciences, Rice University, 6100 Main St MS 126, Houston, Texas 77005, US

Figure S1. Diagram showing the different feedstocks (forest floor and down wood), formation processes (wildfire and slow pyrolysis) and PyC materials used in this study (wildfire charcoals and slow-pyrolysis biochars).

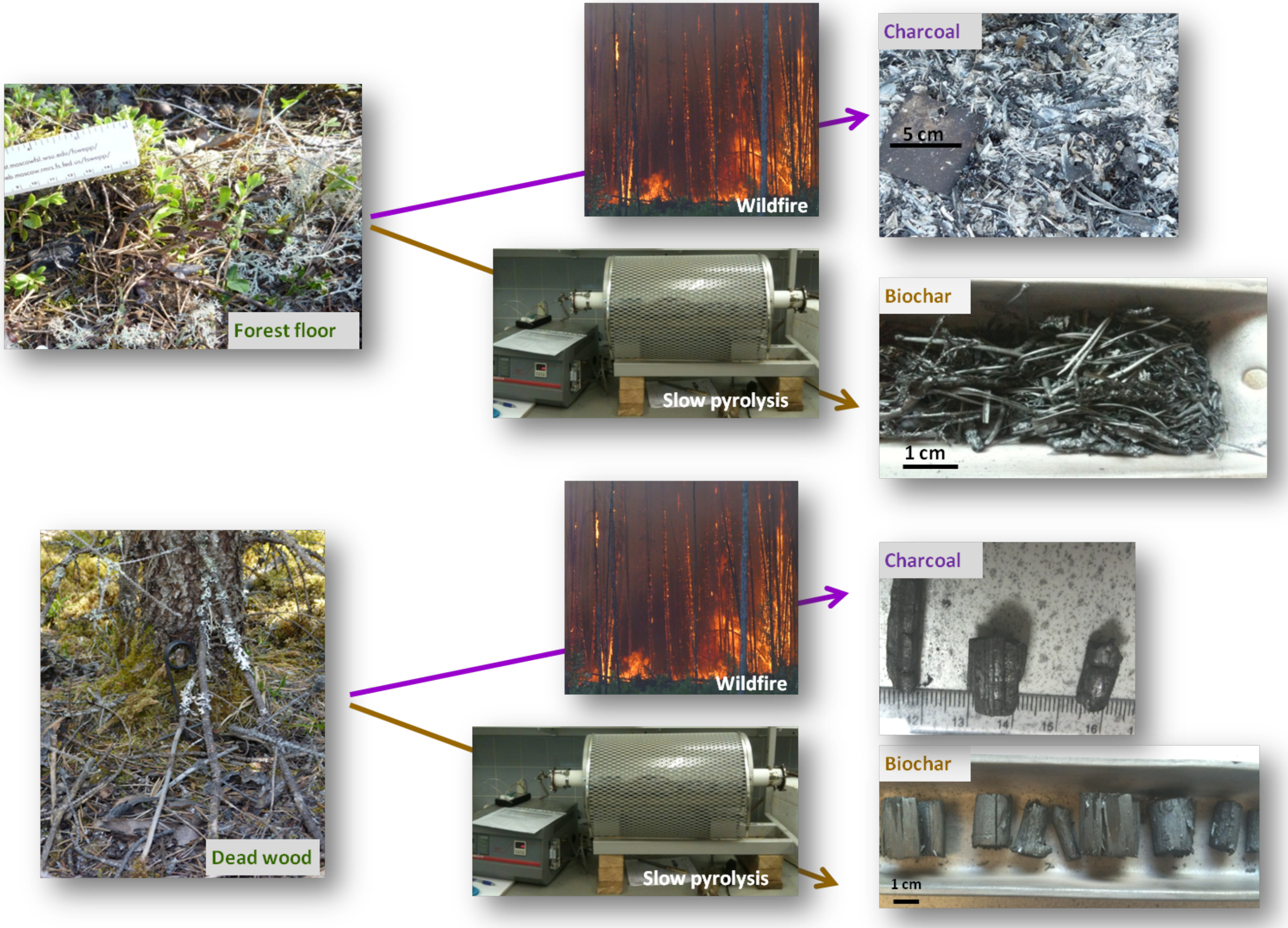

Table S1. Relative concentrations (in %) of the 16 polycyclic aromatic hydrocarbons (PAHs), listed as priority pollutants by the US Environmental Protection Agency (EPA), present in the forest floor (FF) and dead wood (DW) wildfire charcoals and slow-pyrolysis biochars.

	FF charcoal #2	FF charcoal #13	FF charcoal #20	FF biochar #1	FF biochar #2	FF biochar #3	DW charcoal	DW biochar #1	DW biochar #2	DW biochar #3
Naphthalene (%)	59.5	78.4	74.1	70.5	75.8	90.8	59.1	37.8	44.4	71.8
Acenaphthylene (%)	2.6	2.8	2.8	0.2	0.2	0.0	1.6	0.8	0.0	0.0
Acenaphthene (%)	2.5	0.9	1.9	1.3	0.3	0.3	1.2	1.0	11.9	6.0
Fluorene (%)	17.9	1.7	4.8	4.1	2.8	0.9	1.1	5.0	1.0	1.6
Phenanthrene (%)	11.0	10.3	10.6	15.0	9.7	3.9	21.2	16.1	22.0	6.4
Anthracene (%)	0.0	1.0	0.0	1.8	1.7	2.0	1.9	1.8	3.2	11.1
Fluoranthene (%)	1.4	1.5	1.8	1.8	1.2	0.6	2.1	3.9	2.3	1.0
Pyrene (%)	1.5	1.4	1.8	2.0	2.2	0.7	2.9	8.2	2.6	1.0
Benzo[a]anthracene (%)	1.2	0.4	0.5	0.5	1.1	0.2	2.0	2.0	2.9	0.4
Chrysene (%)	1.0	0.8	0.8	0.9	1.2	0.2	2.6	5.9	3.6	0.7
Benzo[b]fluoranthene (%)	0.3	0.2	0.3	0.2	0.4	0.1	0.7	1.9	0.9	0.0
Benzo[k]fluoranthene (%)	0.2	0.1	0.2	0.3	0.2	0.1	0.7	6.6	0.5	0.0
Benzo[a]pyrene (%)	0.6	0.3	0.3	0.6	1.3	0.0	1.6	3.5	2.0	0.0
Indeno[1,2,3-cd]pyrene (%)	0.1	0.1	0.2	0.3	0.6	0.0	0.6	1.9	0.8	0.0
Dibenzo[a,h]anthracene (%)	0.0	0.0	0.0	0.0	0.2	0.0	0.0	1.0	0.2	0.0
Benzo[ghi]perylene (%)	0.2	0.2	0.2	0.5	1.1	0.2	0.8	2.7	1.6	0.0

#: sample number.
